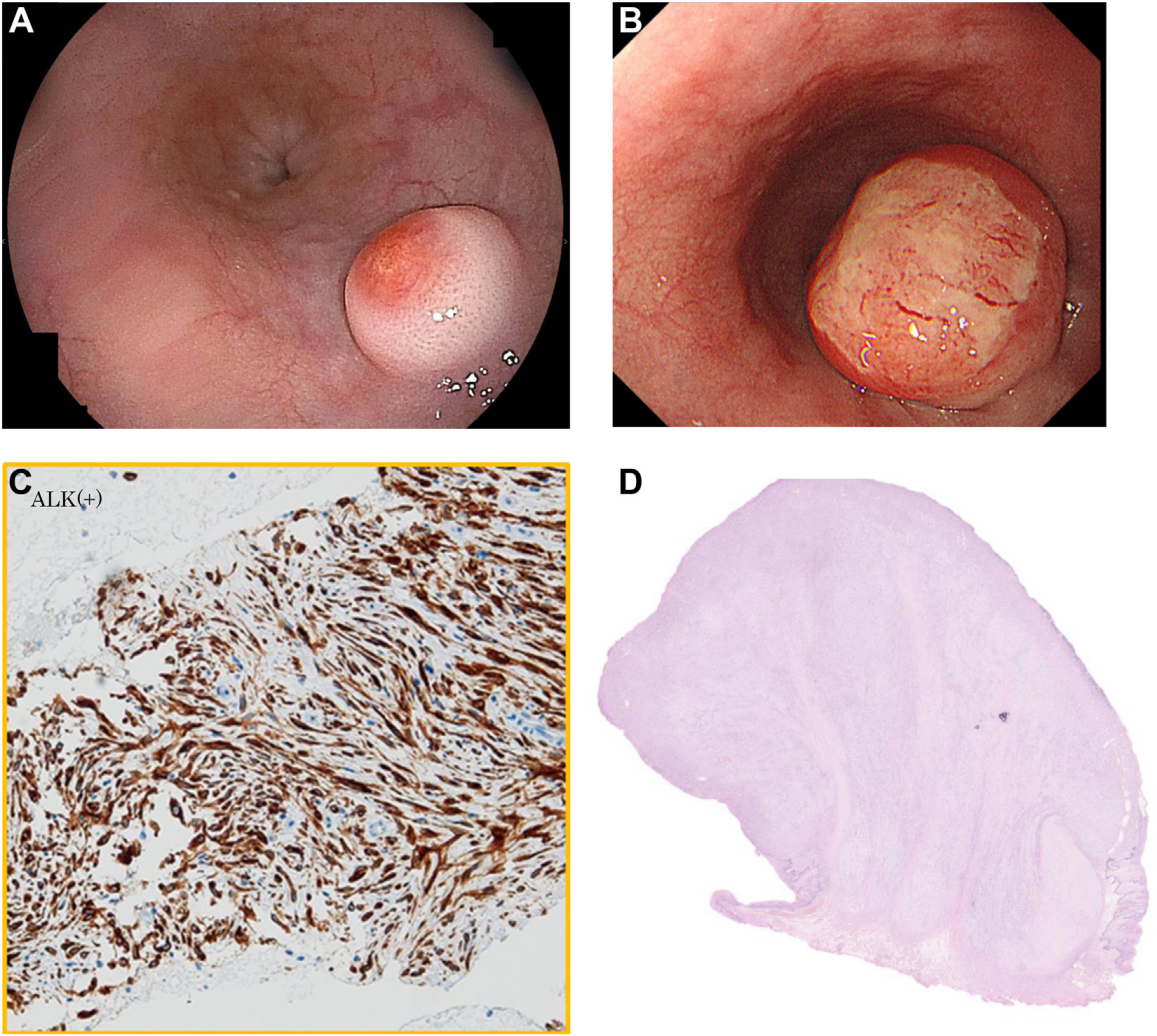# Short-Time Development of Esophageal Inflammatory Myofibroblastic Tumor Which Resected by Endoscopic Submucosal Dissection

**DOI:** 10.1016/j.gastha.2022.02.023

**Published:** 2022-05-06

**Authors:** Sachiyo Onishi, Tsutomu Tanaka, Masahiro Tajika

**Affiliations:** Department of Endoscopy, Aichi Cancer Center Hospital, Nagoya, Japan

A 53-year-old man was pointed out an elevated lesion in the esophagus by screening upper gastrointestinal series. A esophagogastroduodenoscopy showed a submucosal tumor 10 mm in diameter in the lower thoracic esophagus (Figure *A*). A biopsy specimen taken from the tumor showed no malignancy. One year later, the tumor had more than doubled in size (Figure *B*). So, we decided to perform endoscopic ultrasonography–guided fine-needle aspiration for this tumor. Endoscopic ultrasonography showed a hypoechoic mass 21 mm in size which come from the second layer. The biopsy specimen showed spindle cell proliferation and actin and anaplastic lymphoma kinase positivity (Figure *C*), which lead to the diagnosis of inflammatory myofibroblastic tumor (IMT). Because of the strong accumulation on positron emission tomography/computed tomography and the growing trend of submucosal tumor, we decided to perform endoscopic submucosal dissection (ESD). The pathological results were consistent with IMT which complete resected by ESD (Figure *D*). No recurrence has been observed for a year.

Although IMT is a tumor of mesenchymal origin which occurs systemically, there are few reports of its occurrence in the esophagus. This is the first case in which short-time growth can be observed and resected by ESD.

**Figure:**